# Emergent multiferroism with magnetodielectric coupling in EuTiO_3_ created by a negative pressure control of strong spin-phonon coupling

**DOI:** 10.1038/s41467-022-30074-4

**Published:** 2022-05-02

**Authors:** Run Zhao, Chao Yang, Hongguang Wang, Kai Jiang, Hua Wu, Shipeng Shen, Le Wang, Young Sun, Kuijuan Jin, Ju Gao, Li Chen, Haiyan Wang, Judith L. MacManus-Driscoll, Peter A. van Aken, Jiawang Hong, Weiwei Li, Hao Yang

**Affiliations:** 1 MIIT Key Laboratory of Aerospace Information Materials and Physics, College of Physics, Nanjing University of Aeronautics and Astronautics, 211106 Nanjing, China; 2 Jiangsu Key Laboratory of Micro and Nano Heat Fluid Flow Technology and Energy Application, School of Physical Science and Technology, Suzhou University of Science and Technology, 215009 Suzhou, China; 3 School of Aerospace Engineering, Beijing Institute of Technology, 100081 Beijing, China; 4 Department of Physics, Jishou University, 416000 Hunan, China; 5 Max Planck Institute for Solid State Research, Heisenbergstr. 1, 70569 Stuttgart, Germany; 6 Engineering Research Center of Nanophotonics & Advanced Instrument (Ministry of Education), Department of Materials, East China Normal University, 200241 Shanghai, China; 7 Department of Applied Physics, Donghua University, 201620 Shanghai, China; 8 Beijing National Laboratory for Condensed Matter Physics, Institute of Physics, Chinese Academy of Science, 100190 Beijing, China; 9 Center of Quantum Materials and Devices and Department of Applied Physics, Chongqing University, 401331 Chongqing, China; 10 School of materials engineering, Purdue University, West Lafayette, IN 47907 USA; 11 Department of Materials Science and Metallurgy, University of Cambridge, Cambridge, CB3 0FS UK; 12 Present Address: Physical and Computational Sciences Directorate, Pacific Northwest National Laboratory, Richland, WA 99354 USA

**Keywords:** Ferroelectrics and multiferroics, Ferroelectrics and multiferroics, Magnetic properties and materials

## Abstract

Negative pressure has emerged as a powerful tool to tailor the physical properties of functional materials. However, a negative pressure control of spin-phonon coupling for engineering magnetism and multiferroicity has not been explored to date. Here, using uniform three-dimensional strain-induced negative pressure in nanocomposite films of (EuTiO_3_)_0.5_:(MgO)_0.5_, we demonstrate an emergent multiferroicity with magnetodielectric coupling in EuTiO_3_, matching exactly with density functional theory calculations. Density functional theory calculations are further used to explore the underlying physics of antiferromagnetic-paraelectric to ferromagnetic-ferroelectric phase transitions, the spin-phonon coupling, and its correlation with negative pressures. The observation of magnetodielectric coupling in the EuTiO_3_ reveals that an enhanced spin-phonon coupling originates from a negative pressure induced by uniform three-dimensional strain. Our work provides a route to creating multiferroicity and magnetoelectric coupling in single-phase oxides using a negative pressure approach.

## Abstract (web summary)

Negative pressure tailors the physical properties of functional oxide materials. Here, the authors demonstrate an emergent multiferroism with magnetodielectric coupling in EuTiO_3_ created by a negative pressure control of strong spin-phonon coupling.

## Introduction

Owing to the interplay between the fundamental degrees of freedom^1,2^, namely, spin, charge, orbital, and lattice, perovskite oxides exhibit a variety of fascinating functionalities, such as superconductivity, magnetism, ferroelectricity, and multiferroicity. Recently, a negative pressure, due to an enlargement of lattice constants in three-dimensions (3D), has emerged as a powerful tool to tailor and enhance physical properties of known functional oxides, such as ferroelectricity^3–6^ and superconductivity^7^. Density functional theory (DFT) calculations have shown that a negative pressure can increase the tetragonality and the polarization in PbTiO_3_^3^ and BaTiO_3_^8^. Experimentally, Wang et al. demonstrated that a negative pressure can be induced in PbTiO_3_ nanowires through a radial density gradient, and enhanced ferroelectric and piezoelectric properties were observed^4,5^. To obtain a negative pressure, three-dimensional tensile stress needs to be realized in nanoscale regions of the films. However, in standard epitaxial oxide films only in-plane (IP) biaxial strain engineering is possible. A strategy to achieve a negative pressure in epitaxial oxide films involves the growth of self-assembled vertically aligned nanocomposites (VANs) which are composed of two phases^9^. By selecting appropriate two phases with different lattice constants and different elastic moduli, the phase with the stiffer materials can induce a few % tensile strain level at the vertical interfaces. By combining the vertical tensile strain with the horizontal strain induced by the substrate, it is possible to exert a negative pressure on the target phase in the VAN films for tailoring its physical functionalities^10^. Kursumovic et al. showed such negative pressure in BaTiO_3_:Sm_2_O_3_ VAN film^11^. This negative pressure led to the transverse piezoelectric coefficient in the BaTiO_3_ phase, *d*_31_, being positive at a value >200 pm/V and up to at least 250 °C. Zhang et al. utilized the negative pressure to realize a giant remanent polarization of 236.3 *µ*C/cm^2^ in the PbTiO_3_ phase in the PbTiO_3_:PbO VAN film^6^. In YBa_2_Cu_3_O_7-δ_:BaZrO_3_ VAN film, a strong modification of superconducting properties in the YBa_2_Cu_3_O_7-δ_ phase has been achieved by applying a negative pressure^7^. Nevertheless, previous theoretical and experimental works mainly focused on the negative pressure engineering of lattice and orbital degrees for tailoring and enhancing physical properties. The effects of a negative pressure associated with the creation or enhancement of magnetism and multiferroicity are still less explored using a combination of spin, phonon (lattice), and orbital degrees.

The strong cross-coupling of multiple degrees of freedom, such as spin–phonon (lattice) coupling, is a potential source of robust multiferroicity. Amongst perovskite oxides, EuTiO_3_ (ETO) is paraelectric (PE)^12^ and G-type antiferromagnetic (AFM)^13^ with strong spin–phonon coupling and has attracted intensive attention for achieving enhanced ferromagnetic (FM)-ferroelectric (FE) properties and magnetoelectric coupling. In terms of the magnetoelectric properties, DFT calculations have shown how the underlying physics leading to the observation of spin–phonon coupling can be utilized to control the ferroic ground states in ETO^14^. Subsequent experiments in epitaxial strained 22 nm ETO thin films (grown on DyScO_3_ substrates) have demonstrated an IP tensile strain-induced FM–FE phase^15^ and the suppression of the AFM order by an external electric field^16^, both of which are consistent with the theoretical predictions. However, the observed saturation magnetization (~3*µ*_B_/Eu^2+^) is significantly smaller than the theoretical value (~7*µ*_B_/Eu^2+^) because of an inhomogeneous magnetic state with the coexistence of FM and non-FM states formed in ETO thin film^17^. The macroscopic FE polarization and giant magnetoelectric effect have yet to be achieved largely due to the leakage issue in strained ETO thin film with a thickness <25 nm and a lack of high-quality substrates that would provide the necessary value of horizontal biaxial strain. In addition, the microscopic mechanism of magnetic phase transition under epitaxial biaxial strain and magnetoelectric effect induced by the spin–phonon coupling is still under debate^14,18,19^. Back to the spin–phonon coupling in ETO, the most effective path in realizing the magnetoelectric effect is still attributed to the displacement of B-site (Ti) ions along out-of-plane (OOP) direction^16,20^. However, the IP biaxial strain can only indirectly change the Ti displacement via the changed cell volume. Hence, ETO is a unique model system to study how a negative pressure control of strong spin–phonon coupling creates a multiferroicity and magnetoelectric coupling effect.

In this work, we have designed self-assemble VAN thin films composed of ETO and MgO fabricated by using pulsed laser deposition. The results of scanning transmission electron microscopy (STEM) and X-ray diffraction (XRD) show that a uniform 3D epitaxial strain has been implemented to induce a negative pressure in the ETO phase via the MgO phase. This results in a control of strong spin–phonon coupling for creating a FM phase with a Curie temperature (*T*_C_) of ~3.4 K and a FE phase with a *T*_C_ of ~255 K. The interplay between spins and phonons was revealed by measuring the magnetodielectric effect and investigating how the magnetization affects the phonons. DFT calculations unravel how the negative pressure control of the competition of the nearest IP and OOP magnetic interactions and the movement of Ti ions induces ferroic phase transitions and further engineers the strong spin–phonon coupling in ETO. Overall, our work demonstrates a pathway to create a multiferroicity and magnetoelectric effect in single-phase oxides using a negative pressure approach.

## Results

### Structural investigations of an ETO:MgO VAN film by STEM and XRD

**Fig. 1:**
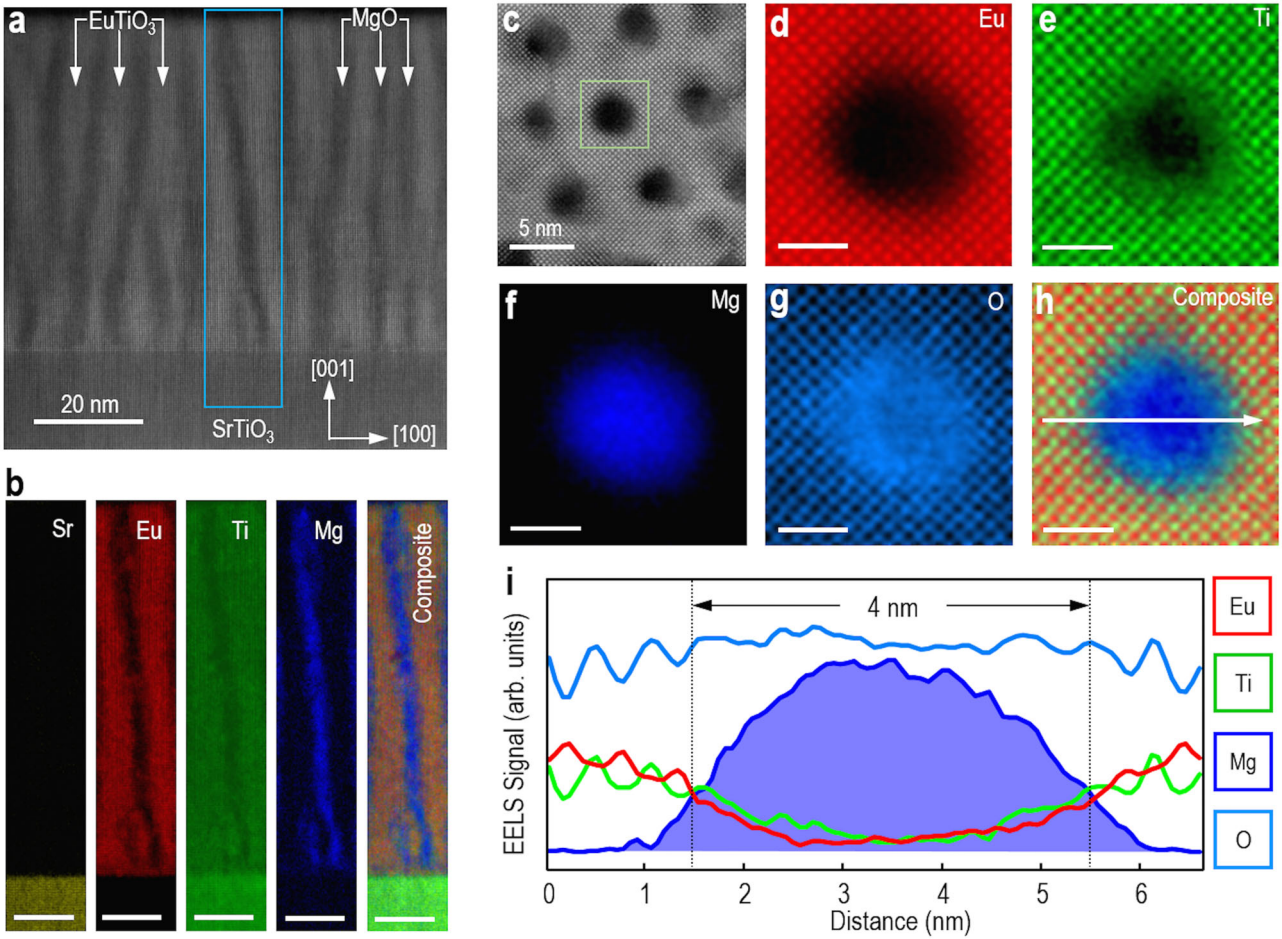
STEM investigations of an ETO:MgO VAN film. **a** Z-contrast HAADF-STEM image of the VAN film in cross-sectional orientation, projected along the [010] zone axis. **b** Atomically resolved STEM-EELS elemental maps of a single nanopillar, corresponding to the rectangle region in (**a**). Scale bar: 10 nm. **c** HAADF-STEM image of the VAN film in plan-view orientation, showing MgO nanopillars embedded in an ETO matrix. **d**–**h** Atomically resolved STEM-EELS elemental maps of a single nanopillar, corresponding to the rectangle region in (**c**). Scale bar: 2 nm. **i** EELS signal intensity profiles across the ETO/MgO interfaces extracted along the white arrow direction in (**h**).

STEM images in the high-angle annular dark-field (HAADF) mode^21,22^, both in cross-section (Fig. 1a) and plan-view (Fig. 1c), as well as STEM electron energy-loss spectroscopy (EELS) maps (Fig. 1b, d–h) show a clear phase separation between high-quality epitaxial ETO and MgO. Vertically aligned interfaces between the two phases form throughout the thin film (Fig. 1a, c). The MgO nanopillars with an average diameter of ~4 nm are uniformly distributed and embedded in an ETO matrix (Fig. 1a, c, Supplementary Fig. 1, and Supplementary Fig. 2c–g). EELS maps in both the cross-section (Fig. 1b) and plan-view images (Fig. 1d–i) orientations show that the interface between the two phases in the VAN is very sharp, as expected, since the structure forms by self-assembly.

The epitaxial quality of ETO:MgO VAN films was further studied by XRD *θ*−2*θ* scans (Supplementary Fig. 2a). The films show sharp (00*l*) peaks of ETO and MgO, indicative of a preferential orientation of the two phases. The OOP lattice constant of ETO is calculated to be 4.01 ± 0.01 Å (Supplementary Table 1). Comparing with bulk ETO lattice constant of 3.905 Å, the corresponding vertical strain (along the c-axis) is calculated to be +2.56% for the ETO phase, which results from the large vertical interfacial area and the lattice mismatch between the two phases in the VAN film.

### 3D strain-induced negative pressure in an ETO:MgO VAN film

**Fig. 2:**
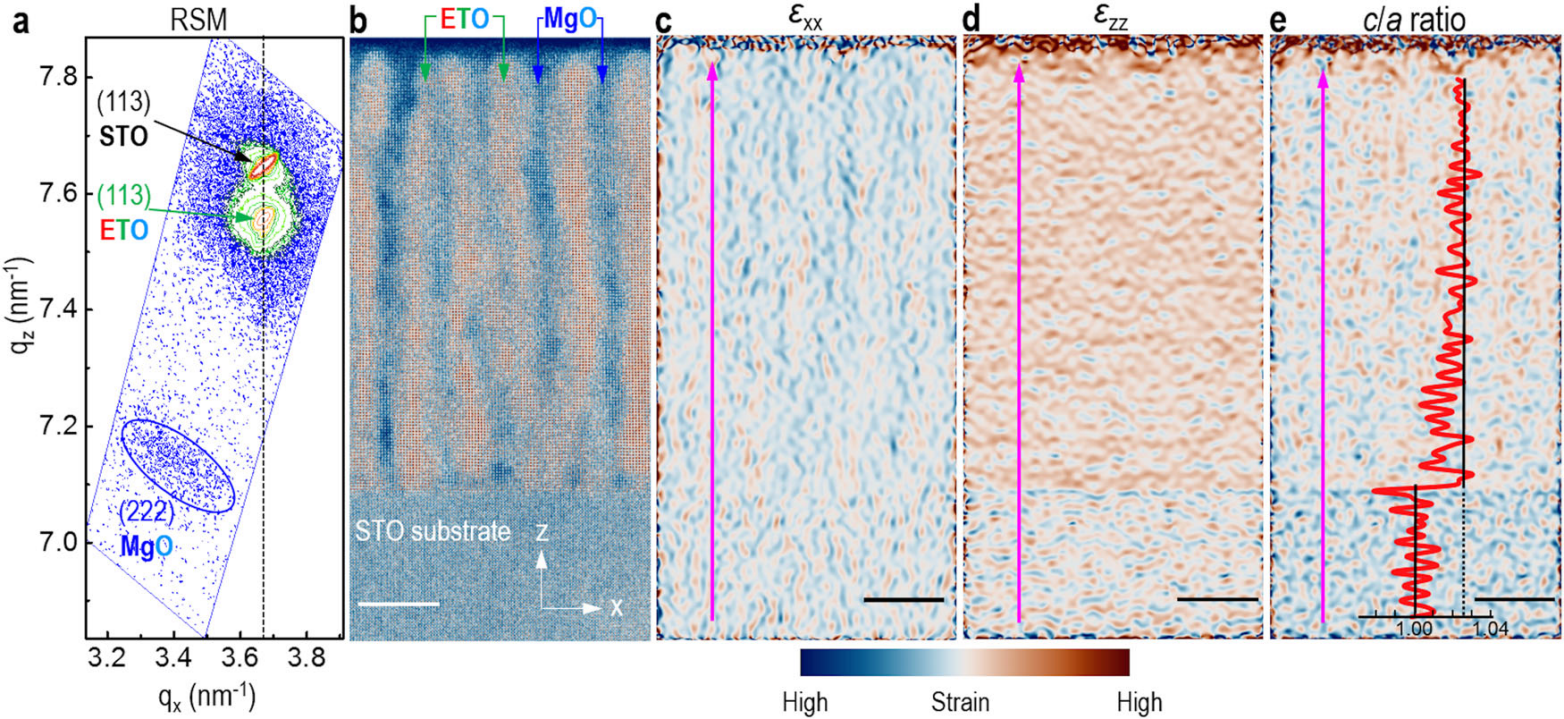
Strain analysis of an ETO:MgO VAN film. **a** Reciprocal space map of (113) Bragg reflection of ETO and STO, and (222) Bragg reflection of MgO. **b** Colored HAADF-STEM image of the VAN film in cross-section. Respective IP (*ε*_xx_) (**c**) and OOP (*ε*_zz_) (**d**) strain maps obtained from STEM-based GPA. The lattice of the STO substrate was taken as a reference for strain analysis. **e** The corresponding *c*/*a* map calculated with (**c**, **d**). The inset shows a profile of *c*/*a* ratio extracted along the arrow direction. Scale bar: 20 nm.

To understand more about how a negative pressure forms in the VAN film, the growth and lattice matching modes of the VAN film were revealed via the close-up STEM image of an individual MgO nanopillar (Supplementary Fig. 3). Owing to the structural misfit and thermal expansion effect in the nucleation stage, the isolated MgO (rock salt) islands wet the STO (perovskite) substrate and are embedded in the ETO film matrix (region 1 in Supplementary Fig. 3a). For a VAN film thickness above 5 nm, the MgO nanopillars developed and then the vertical interfaces between ETO and MgO were formed along the OOP direction (region 2 in Supplementary Fig. 3a). During the growth of the VAN film, the ETO phase experienced a volume expansion in three mutually perpendicular directions, which is induced by that the stiffer STO substrate and the MgO nanopillars impart a negative pressure on the ETO (*C*_11-STO_»*C*_11-ETO_ and *C*_44-MgO_ > *C*_44-ETO_)^23,24^. Under such negative pressure, the embedded MgO nanopillars exert a *tensile* force on the surrounding ETO phase along the OOP direction due to the lattice mismatch between ETO and MgO. The MgO nanopillars along the IP direction are not constrained by the substrate and it undergoes compression from the expanded ETO matrix upon cooling due to thermal expansion effects^25^. Misfit dislocations were formed at the vertical interfaces between the ETO and MgO phases for relaxing the misfit strain (Supplementary Fig. 4). Furthermore, a more detailed analysis of the crystal structure was undertaken by high-resolution asymmetric X-ray reciprocal space map (RSM) around the (113) and (103) reflections of STO. An enhanced OOP lattice constant in the ETO phase (4.01 ± 0.01 Å) indicates that the ETO phase is OOP tensed by the MgO phase (the bulk average lattice constant of ETO is 3.905 Å, and of MgO is 4.212 Å). As shown in Fig. 2a, the STO and ETO peaks share the same *q*_*x*_ position, indicating that the ETO phase in the VAN film is coherently strained to the STO substrate. Hence, 3D strain is formed in the ETO phase. And IP lattice constant of the ETO phase is determined to be 3.90 ± 0.01 Å (Table S1). Based on the IP (*a*) and OOP (*c*) lattice constants of ETO, a *c*/*a* ratio of 1.03 is obtained. Considering that the elastic constant *C*_44_ of ETO phase is 116.3 GPa under zero pressure^23^, a negative pressure along the OOP direction (for the measured *c/a* = 1.03) is estimated to be around −2.98 GPa. In addition, there is no difference in the (103), (013), (−103), and (0–13) Bragg peak positions of the ETO phase (Supplementary Fig. 5), indicating that the ETO phase has a tetragonal structure. The angles between TiO_6_ octahedra in the ETO phase are further found to be around 180° along the IP and OOP directions (Supplementary Fig. 6 and Supplementary Fig. 7). Hence, according to the Glazer notation^26^, the space group of the ETO phase is determined to be tetragonal *P*4/*mbm* (*a*^0^*a*^0^*c*^+^ or *a*^0^*a*^0^*c*^−^)^27^.

The distribution of 3D strain in the ETO phase was further visualized by conducting geometric phase analysis (GPA) from the HAADF-STEM image (Fig. 2b)^28^. The IP strain component (*ɛ*_xx_) and OOP strain component (*ɛ*_zz_) calculated relative to the STO substrate region are shown in Fig. 2c–e. These maps clearly demonstrate that the ETO phase in the VAN film has a very uniform 3D strain distribution. More importantly, a profile of *c*/*a* values is extracted along the arrow direction, as shown in Fig. 2d, and an average *c*/*a* ratio is around 1.03, agreeing very well with the value determined from XRD and RSM measurements.

### Multiferroic properties of an ETO:MgO VAN film

**Fig. 3:**
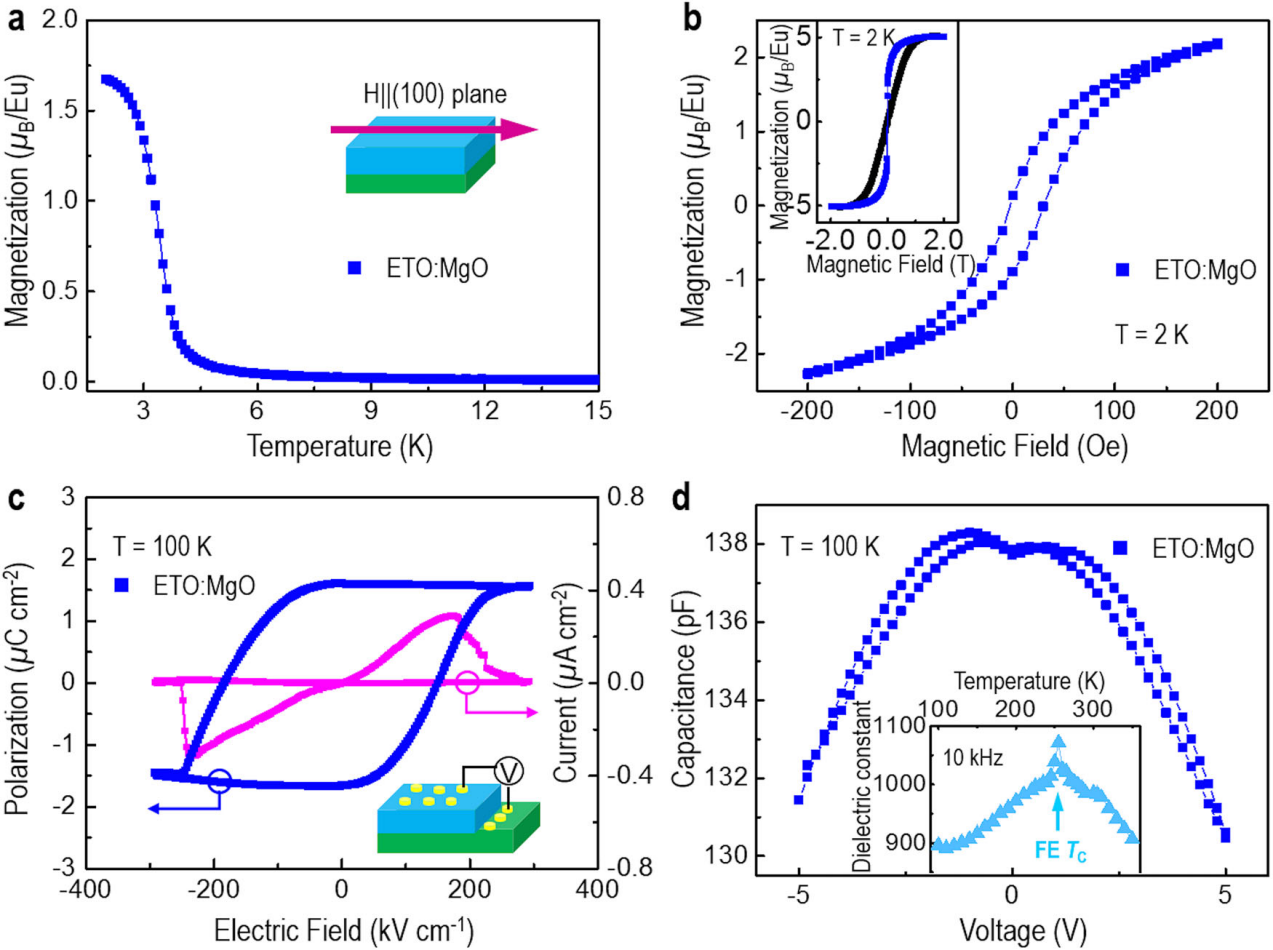
Multiferroic properties of an ETO:MgO VAN film. **a** Temperature and (**b**) magnetic field-dependent magnetization measured by applying a magnetic field along the IP direction. The inset of (**b**) shows the comparison of magnetic hysteresis loops measured by applying a magnetic field along the IP (blue) and OOP (black) directions. **c** Ferroelectric polarization hysteresis loop and switching current measured by PUND and at 1 kHz. **d** Capacitance–voltage curve measured at 100 K. The inset shows the temperature-dependent dielectric constant showing a FE *T*_C_ of ∼255 K.

The magnetic properties of 300 nm thick VAN and 300 nm thick plain films are compared in magnetization versus temperature (*M*–*T*) and magnetic field (*M*–*H*) plots in Fig. 3a, b and Supplementary Fig. 8. The plain ETO film displays a distinct AFM transition around 5.1 K (Supplementary Fig. 8b), close to magnetic ordering temperature (*T*_N_ ~ 5.3 K) for bulk ETO^12,13^. In contrast, the magnetization (*M)* of the VAN film increases monotonically with decreasing temperature and almost saturates at 2 K (Fig. 3a). Furthermore, a clear magnetic hysteresis loop with a coercivity (*H*_C_) of ∼18 Oe is observed in the VAN film. Hence, these results strongly suggest that the ETO phase is FM in the VAN film. The FM Curie temperature (*T*_C,FM_) is further determined to be ~3.4 K (Supplementary Fig. 8d). The saturation magnetization (*M*_S_) value measured at ± 2 T is around 5 *µ*_B_/Eu^2+^, which is much higher than that of the strained 22 nm ETO thin film grown on the DyScO_3_ substrate (~3*µ*_B_/Eu^2+^)^13^. As we show later, this can be attributed to the formation of a homogeneous magnetic state induced by uniform 3D strain in the VAN film.

Strong ferroelectric (FE) properties were observed in the 300 nm-thick ETO:MgO VAN film (Fig. 3c, d). After subtracting the linear dielectric contribution, a polarization–electric field (*P–E*) hysteresis loop with a switched polarization (∆*P*) of value ~3.3 *µ*C/cm^2^ and switching current were obtained at 100 K (Fig. 3c). Using the Berry phase methods^29^, the ferroelectric polarization was further calculated (Supplementary Fig. 9). Its value is of the same order of magnitude with the experimental value (Supplementary Fig. 10). This implies that the ETO phase is FE in the VAN film. A capacitance–voltage (*C–V*) hysteresis curve was also measured at 100 K (Fig. 3d). The observation of a butterfly-like characteristic shape of *C–V* hysteresis curve further confirms that the ETO phase is FE.

To determine the FE Curie temperature (*T*_C,FE_), the temperature-dependent dielectric constant was measured at a frequency of 10 kHz and is shown as an inset of Fig. 3c. The peak position of PE to FE is clearly visible at ~255 K, comparable to the previous work^15^. The peak position does not shift with frequency (Supplementary Fig. 11), indicating a normal, rather than a relaxed, FE transition formed in the ETO phase^30^. It should be pointed out that, due to the low dielectric constant of MgO, its contribution to the dielectric constant of the VAN film can be neglected^31^. Overall, the above results show that the ETO phase in the VAN film is transformed from PE in bulk ETO into FE to at least at 100 K, with a *T*_C,FE_ of around 255 K.

## Discussion

**Fig. 4:**
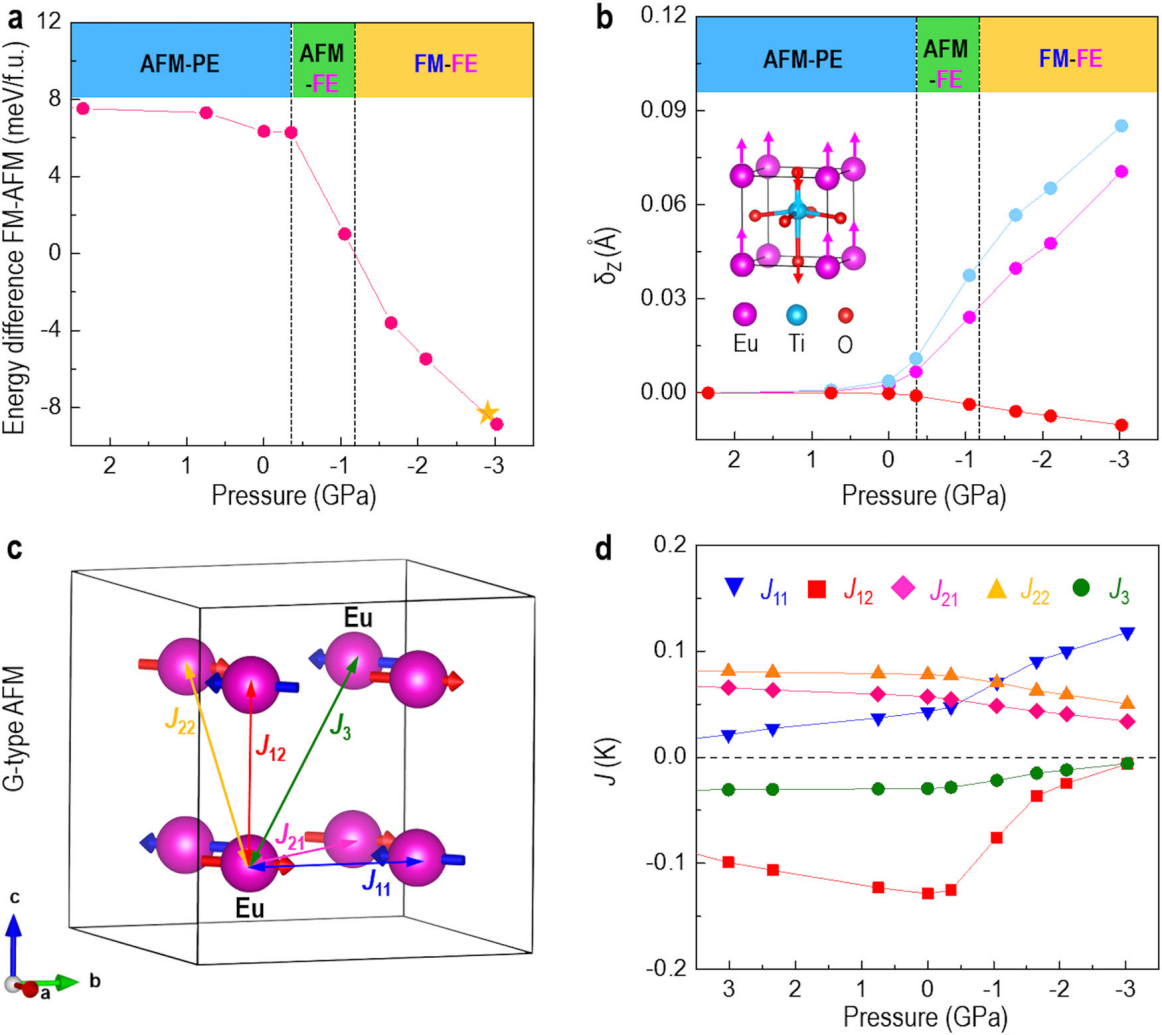
Phase transitions and magnetic interactions in ETO under pressure, from DFT calculations. **a** The energy difference between FM and AFM showing a clear magnetic phase transition. The star represents the experimental negative pressure. **b** Atom displacement along z-direction (OOP direction) showing a clear ferroelectric phase transition. The inset shows a schematic diagram for the movement of atoms under pressure. **c** Schematic diagram with a G-type AFM magnetic configuration adopted for ETO, with different interactions. *J*_*1m*_ (*m* = 1,2) refers to the nearest interactions, *J*_*2n*_ (*n* = 1,2) refers to the next-nearest interactions, and *J*_*33*_ refers to the next-next-nearest interactions. **d** Calculated variation of magnetic exchange coefficients of ETO under different pressures.

To gain further insights into the correlation between negative pressure and its link to the multiferroic properties and electronic structures in ETO, we performed the first-principles calculations. The simulated energy difference between FM and AFM configurations clearly shows a magnetic phase transition from AFM to FM at a negative pressure of ~−1.17 GPa (Fig. 4a). In addition, the extracted atom displacements (Fig. 4b) show that the oxygen atoms move downward, while the Eu and Ti atoms move upward along the OOP direction under a negative pressure. Overall, the ETO experiences three types of phase transitions with the increase of negative pressure along the OOP direction, i.e., AFM–PE ground phase, AFM–FE phase with a negative pressure larger than ~−0.35 GPa, and FM–FE phase with a negative pressure larger than ~−1.17 GPa. Since the movement of atoms affects the relative strength of the atomic orbital interactions, it is expected that magnetic exchange coupling (Fig. 4c) will be changed as well^32–34^. As shown in Fig. 4d, the nearest OOP AFM (*J*_*12*_) dominates the magnetic coupling and determines an AFM ground state at ambient pressure. With the increase of negative pressure, the exchange coefficients of *J*_*12*_, *J*_*21*_, *J*_*22*_, and *J*_*3*_ reduce sharply and *J*_*12*_ and *J*_*3*_ are close to zero at ~−3 GPa. In contrast, the nearest IP magnetic interaction *J*_*11*_ increases quickly and its absolute value becomes the largest one compared with the other magnetic interactions. Hence, the AFM to FM phase transition mainly originates from the competition between the AFM exchange interaction *J*_*12*_ and FM exchange interaction *J*_*11*_ under negative pressures, due to stronger interactions between Eu 4 *f*, O 2*p*, and Ti 3*d* orbitals (Supplementary Fig. 12). This is quite different from the AFM to FM phase transition in IP biaxial strained ETO, in which the competition between the AFM superexchange and the indirect FM exchange via Eu 5*d* states^18^, or the modification of the third nearest-neighbor Eu ion superexchange interaction mediated through the central Ti ion^16^.

**Fig. 5:**
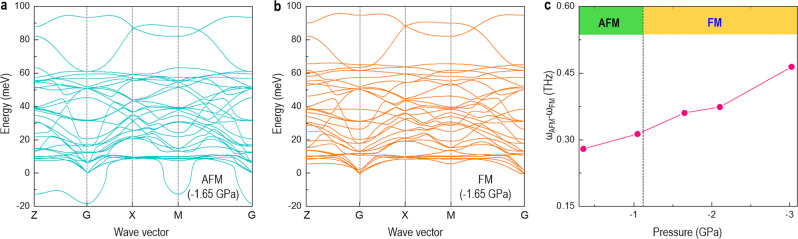
Phonon dispersions in ETO under negative pressure, from DFT calculations. **a** Phonon dispersion in ETO with an AFM configuration and ideal crystal structure. **b** Phonon dispersion in ETO with a FM configuration and distorted FE crystal structure. **c** The change of soft phonon frequency (polar mode) of ETO with a distorted FE crystal structure under different negative pressures.

It shows that the FM interactions dominate the magnetic coupling, when a negative pressure is larger than ~−1.17 GPa, agreeing well with the observation of FM in the ETO phase in VAN film under an experimental negative pressure of −2.98 GPa. Additionally, the magnetic exchange coefficient of *J*_*12*_ experiences an abrupt change at ~−0.35 GPa. This value is coincided with the critical pressure, where the PE-FE phase transition occurs. This indicates that the magnetic spins are coupled with phonons (lattice) in ETO. As further evidenced in Fig. 5a, the phonon dispersion of ETO with an AFM configuration possess large negative modes at **Z**, **G**, and **M** high symmetry points. In contrast, at the same negative pressure of −1.65 GPa, the calculated phonon dispersions of ETO with a FM configuration have no negative modes (Fig. 5b). Furthermore, upon applying a negative pressure, the largest frequency shift (Fig. 5c) is around 0.48 THz which is comparable to the reported values in other multiferroic materials^35,36^. It should be noticed that the ferroelectric phase transition also affects the soft mode. These results confirm that spin and phonons are strongly coupled, which can be tuned by a negative pressure simultaneously.

**Fig. 6:**
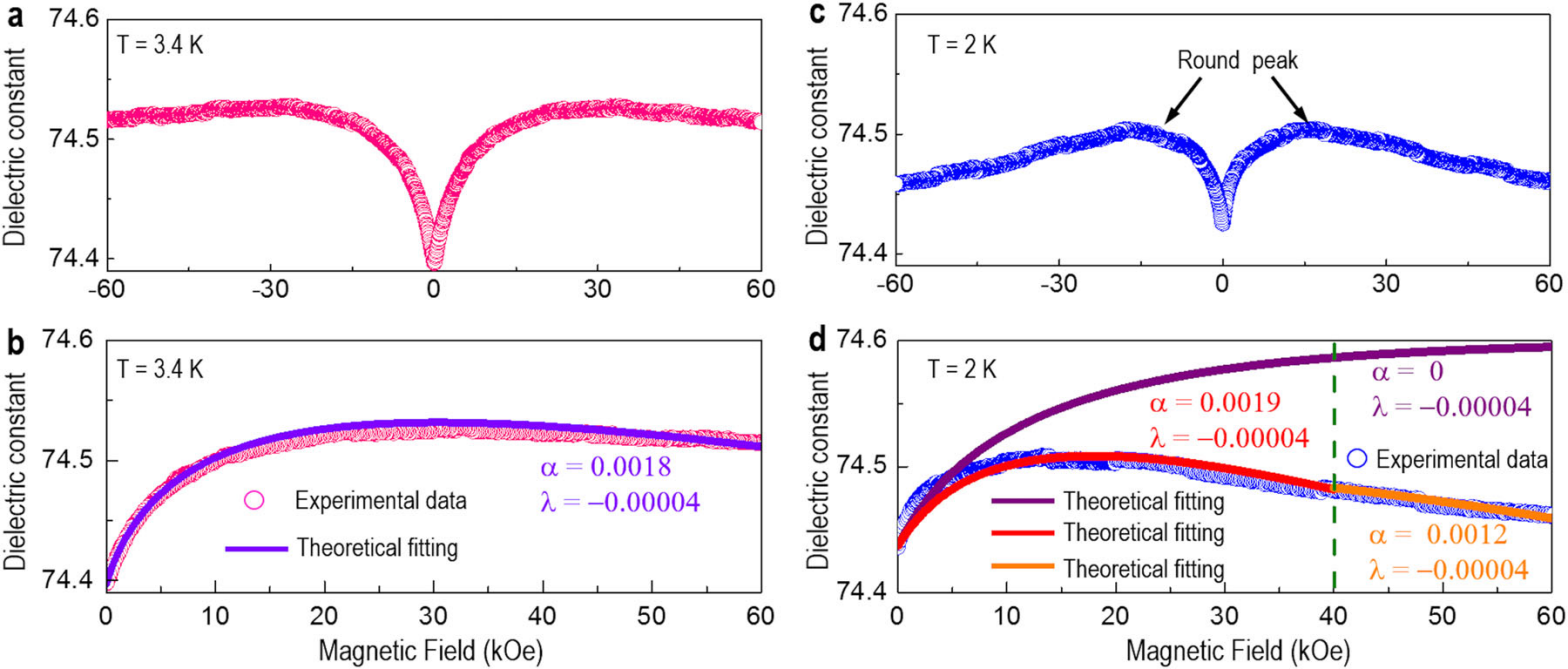
Magnetodielectric coupling of an ETO:MgO VAN film. Magnetic field-dependent dielectric constant measured at 3.4 K (**a**) and 2 K (**c**). Comparison of the theoretical results with the experimental data of the magnetic field-dependent dielectric constant at 3.4 K (**b**) and 2 K (**d**). The open circles represent the experimental data, while the solid lines represent the theoretical results with different parameters. *λ* is the biquadratic spin–phonon coupling coefficient and *α* is the corresponding coefficient to scale the strength of the tuning effect of the magnetic field on the lattice parameters.

Owing to the spin–phonon coupling significantly modified by the negative pressure, magnetoelectric coupling was further achieved by performing the magnetodielectric measurements (Fig. 6a, c and Supplementary Fig. 13). When the temperature is 10 K, the ETO is in its PM-FE state (Supplementary Fig. 14). The magnetization increases with the magnetic field. Due to the spin–phonon interactions, the dielectric constant monotonically increases with magnetic field, following a behavior similar to that of the magnetization versus magnetic field^12,20^. Interestingly, with a further decrease of temperature to 3.4 K (Fig. 6a) and 2 K (Fig. 6c), as ETO transforms into its FM–FE state, the dielectric constant as a function of the magnetic field evidently deviates from the monotonically increasing trend, showing a round peak at ± 16 kOe (Fig. 6c).

In order to capture the underlying physics more clearly, we performed the Landau free energy expansion (Supplementary Discussion 1) to describe the coupling between the spin (magnetic ordering parameters) and phonon (lattice). When the temperature is at 3.4 K, the round peak in the magnetic field dielectric constant is negligible (Fig. 6a). The magnetic moments tend to be disordered at the magnetic phase transition temperature of *T*_C,FM_ (3.4 K), so the magnetic field plays a more significant role in ordering magnetic moments. Consequently, this causes a more remarkable increase of the dielectric constant with an increasing magnetic field. Meanwhile, by fitting the experimental data (Fig. 6b), the coupling strength α between the magnetic field and the strain is found to decrease in comparison to 2 K. As a result, the effect arising from the magnetic field on the alignment of the magnetic spins and enhancement of the dielectric constant is strong enough to suppress the reduction of the dielectric constant that is caused by the magnetostriction^37^, leading to a disappearance of the round peak at 10 K (Supplementary Fig. 14).

As the temperature decreases to 2 K, the ETO is in a FM–FE state, i.e. both *M*_S_ and *P*_S_ are nonzero and these two order parameters are coupled to each other. Considering the magnetic field-induced magnetostriction, which contributes to the stabilization of the FE phase and reduces the dielectric susceptibility in the FE phase, the dielectric susceptibility gradually bends from its monotonic increasing trend as the magnetic field increases and shows a round peak (Fig. 6c). By fitting the experimental data with different types of theoretical models (Fig. 6d), we find that the competition between the two mechanisms, i.e. spin–phonon coupling and magnetostriction, causes the unusual behavior of dielectric constant with magnetic field. A spin–phonon coupling at a lower magnetic field and a magnetostriction at the middle range of a magnetic field can be obtained. Up to a higher magnetic field of 40 kOe, weakened magnetostriction arises from the crossover from a negative to a positive increased length (Supplementary Discussion 2).

In summary, in this study, epitaxial ETO:MgO VAN thin films were designed to explore a negative pressure control of strong spin–phonon coupling for creating multiferroicity and magnetoelectric coupling in the single-phase ETO. Vertical epitaxy along with the sharp MgO/ETO interfaces produces an enormous tetragonality (*c*/*a* = 1.03) in the ETO phase. The calculated negative pressure along the OOP direction yields −2.98 GPa. Consequently, this enables the transformation of the ETO phase from bulk AFM–PE into FM–FE with corresponding *T*_C_s of 3.4 K and 255 K, respectively. Due to the formation of a large negative pressure induced by uniform 3D strain, a pure *P*_S_ value of 1.65 *µ*C/cm^2^ with OOP polarization is achieved. DFT calculations reveal that under the negative pressures the magnetic phase transition is mainly due to the competition between the AFM exchange interaction *J*_*12*_ and FM exchange interaction *J*_*11*_ and the spins are strongly coupled with phonons. Furthermore, due to the strong spin–phonon coupling, the magnetoelectric coupling effect is achieved in the FM–FE state of ETO. This work presents a promising approach to negative-pressure-engineered single-phase 3*d* or 5*d*-based multiferroics^38^, originating from the unique structure and properties of the VAN films.

## Methods

### Thin film preparation

Self-assembled VAN thin films of (EuTiO_3_)_0.5_:(MgO)_0.5_ were grown on both (001) SrTiO_3_ (STO) and 0.5 wt.% Nb-doped SrTiO_3_ (Nb-STO) substrates by pulsed laser deposition. Film thicknesses of 70 nm and 300 nm were grown. As a reference, a plain EuTiO_3_ film of 300 nm thickness was grown on a LaAlO_3_ (LAO) substrate (Supplementary Fig. 15). All thin films were grown at a deposition temperature of 750 °C with an oxygen pressure of 5 × 10^−3^ Pa and then cooled down to room temperature under the same oxygen pressure. The laser pulse rate was 3 Hz and the laser fluence was 2 J/cm^2^.

### XRD characterization

The phase and the crystalline quality of the thin films were investigated by X-ray diffraction (XRD) on a high-resolution X-ray diffractometer (Empyrean, PANalytical, The Netherlands) using Cu K_α_ radiation (*λ* = 1.5405 Å). To explore the 3D strain state, asymmetric RSM around (113) of STO was also measured. IP (*a* and *b*) lattice parameters were determined by RSM map using the epitaxy software package. The *c* lattice parameter was calculated from theta-2theta, was also cross-checked by the RSM map and was found to be the same within ±0.01 Å.

### Ferroelectric, dielectric, magnetic and magnetodielectric characterizations

Ferroelectric hysteresis loops and switching current were collected from the current density–voltage (*J–V*) curves via the double-wave method in a physical properties measurement system (PPMS-9, Quantum Design)^39,40^. Dielectric measurements were carried out using an Impedance analyzer (E4990A). The temperature was controlled by a Linkam Scientific Instruments HFS600E-PB4 system. The magnetodielectric effects were measured using an Agilent 4980 A LCR meter in a cryogen-free superconducting magnet system (Oxford Instruments, TeslatronPT). For the ferroelectric, dielectric, and magnetodielectric measurements, Pt top electrodes with an area of 8 × 10^−4^ cm^2^ and the VAN films grown on Nb-STO substrates were used. The magnetic measurements were carried out using a PPMS-9 and a superconducting quantum interface device magnetometer (SQUID, Quantum Design).

### Scanning transmission electron microscopy

STEM specimens in both cross-sectional and plan-view orientations were prepared by tripod polishing followed by argon-ion milling at liquid-N_2_ temperature^41^. STEM studies were conducted using a spherical aberration-corrected STEM (JEM-ARM200F, JEOL Co. Ltd.) equipped with a cold-field emission gun and a DCOR probe Cs-corrector (CEOS GmbH) operated at 200 kV. The STEM images were obtained by an ADF detector with a convergent semi-angle of 20.4 mrad and collection semi-angles of 70−300 mrad. In order to make precise measurements of lattice constants, ten serial frames were acquired with a short dwell time (2 μs/pixel), aligned, and added afterward to improve the signal-to-noise ratio (SNR) and to minimize the image distortion of HAADF images. Atomically resolved HAADF-STEM images have been analyzed using Geometric Phase Analysis (GPA) for strain analysis. EELS acquisition was performed by a Gatan GIF Quantum ERS imaging filter equipped with a Gatan K2 Summit camera with a convergent semi-angle of 20.4 mrad and a collection semi-angle of 111 mrad. EELS spectrum imaging was performed with a dispersion of 0.5 eV/channel and 456 eV drift tube energy with a 4000-pixel wide detector for the simultaneous acquisition of signals of Ti-L_2,3_, O-K, Eu-M_4,5_, Mg-K, and Sr-L_2,3_ edges. Energy-dispersive X-ray spectroscopy (EDS) was performed using a 100 mm^2^ JEOL Centurio SDD-EDX detector. The raw spectrum image data were denoised by applying a principal component analysis (PCA) with the multivariate statistical analysis (MSA) plugin (HREM Research Inc.) in Gatan DigitalMicrograph and then smoothed using a spatial filter in Gatan DigitalMicrograph. The analysis of the size distribution of MgO pillars was conducted using a built-in function in Gatan DigitalMicrograph. The coordinate positions of oxygen ions were determined by Gaussian fitting the oxygen columns in ABF images, and then used to calculate the angles between neighboring TiO_6_ octahedra^42,43^.

### Density functional theory

We performed DFT calculations in the Vienna ab initio simulation package (VASP) with projector-augmented wave potentials in the GGA + U framework^44,45^. An on-site Coulomb parameter *U* = 5.7 eV and Hund’s exchange *J* = 1.0 eV were chosen for the strong electron correlation effects on the *f* shells of Eu atom, which were well tested in the work of Lee et al.^15^. The *I*4/*mcm* structure with the lowest total energy was chosen as the ground state, which was confirmed by previous experiment work^46^. The 6 × 6 × 6 k-point grid and a 500 eV plane-wave energy cutoff were used in our calculations. Geometric relaxations were done by keeping the IP lattice parameter fixed and relaxing the OOP lattice parameters. Residual force threshold was set to 0.001 eVÅ^−1^. Exchange parameters for an Ising model were fitted to total energy calculations in a 2 × 2 × 2 perovskite supercell that consists of 40 atoms and 8 different magnetic configurations. Different from the exchange constants for *c*/*a* ~ 1 structures^35,36^, a total of five magnetic exchange constants are considered in our calculations due to the experimental ratio of *c*/*a* ~ 1.03. The details of defined and calculated magnetic exchange constants can be found in Supplementary Discussion 3. We calculated the phonon frequency with the help of Phonopy package and the DFPT method implemented in the VASP code.

### Reporting summary

Further information on research design is available in the Nature Research Reporting Summary linked to this article.

## Supplementary information

Supplementary information

Reporting Summary

Lasing Reporting Summary

## Data Availability

All the experimental/calculation date that support the findings of this study are available from the corresponding authors upon reasonable request.

## Code availability

All the codes used for this study are available from the corresponding authors upon reasonable request.
