# Supplementary material for: Emergent multiferroism with magnetodielectric coupling in EuTiO3 created by a negative pressure control of strong spin-phonon coupling
Source: Nat Commun. 2022 May 2;13:2364. doi: 10.1038/s41467-022-30074-4 (PMC9061821; doi:10.1038/s41467-022-30074-4)
Supplement: Supplementary file 1 — Supplementary information [file 41467_2022_30074_MOESM1_ESM.pdf]

Supplementary information

Emergent multiferroism with magnetodielectric coupling in $EuTiO_3$ created by a negative pressure control of strong spin-phonon coupling

Run Zhao[1,2,†], Chao Yang[3,11,†], Hongguang Wang[4,*], Kai Jiang[5,*], Hua Wu[6], Shipeng Shen[7], Le Wang[7,#], Young Sun[8], Kuijuan Jin[7], Ju Gao[2], Li Chen[9], Haiyan Wang[9], Judith L. MacManus-Driscoll[10], Peter A. van Aken[4], Jiawang Hong[3,*], Weiwei Li[1,10*] and Hao Yang[1,*]

1. MIIT Key Laboratory of Aerospace Information Materials and Physics, College of Physics, Nanjing University of Aeronautics and Astronautics, Nanjing 211106, China
2. Jiangsu Key Laboratory of Micro and Nano Heat Fluid Flow Technology and Energy Application, School of Physical Science and Technology, Suzhou University of Science and Technology, Suzhou 215009, China
3. School of Aerospace Engineering, Beijing Institute of Technology, Beijing 100081, China
4. Max Planck Institute for Solid State Research, Heisenbergstr. 1, 70569, Stuttgart, Germany
5. Engineering Research Center of Nanophotonics & Advanced Instrument (Ministry of Education), Department of Materials, East China Normal University, Shanghai 200241, China
6. Department of Applied Physics, Donghua University, Shanghai 201620, China
7. Beijing National Laboratory for Condensed Matter Physics, Institute of Physics, Chinese Academy of Science, Beijing 100190, China
8. Center of Quantum Materials and Devices and Department of Applied Physics, Chongqing University, Chongqing 401331, China
9. School of materials engineering, Purdue University, West Lafayette, IN, 47907, USA
10. Department of Materials Science and Metallurgy, University of Cambridge, Cambridge CB3 0FS, UK
11. Department of Physics, Jishou University, Hunan 416000, China

Corresponding authors: hgwang@fkf.mpg.de, kjiang@ee.ecnu.edu.cn, hongjw@bit.edu.cn, wl337@nuaa.edu.cn, yanghao@nuaa.edu.cn

[†]These authors contribute equally to this work

[#]Present address: Physical and Computational Sciences Directorate, Pacific Northwest National Laboratory, Richland, Washington 99354, USA

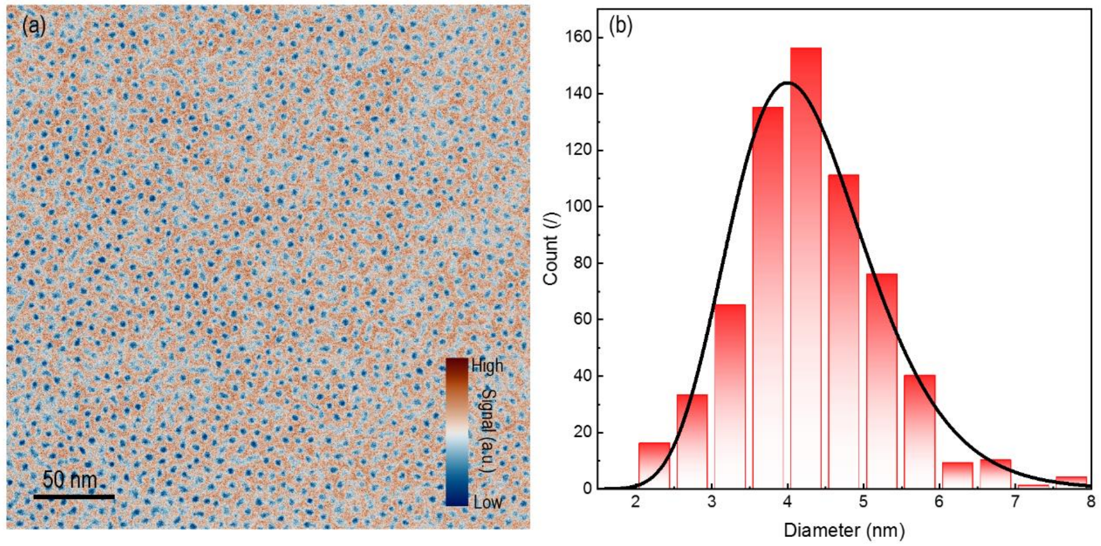

Supplementary Figure 1 STEM investigation of an ETO:MgO VAN film. (a) HAADF-STEM image of an ETO:MgO VAN film in plan-view orientation, showing MgO nanopillars (dark spots) embedded in an ETO matrix. (b) Size distribution of the MgO nanopillars in (a), indicating that the diameter of MgO nanopillars is mostly about 4 nm.

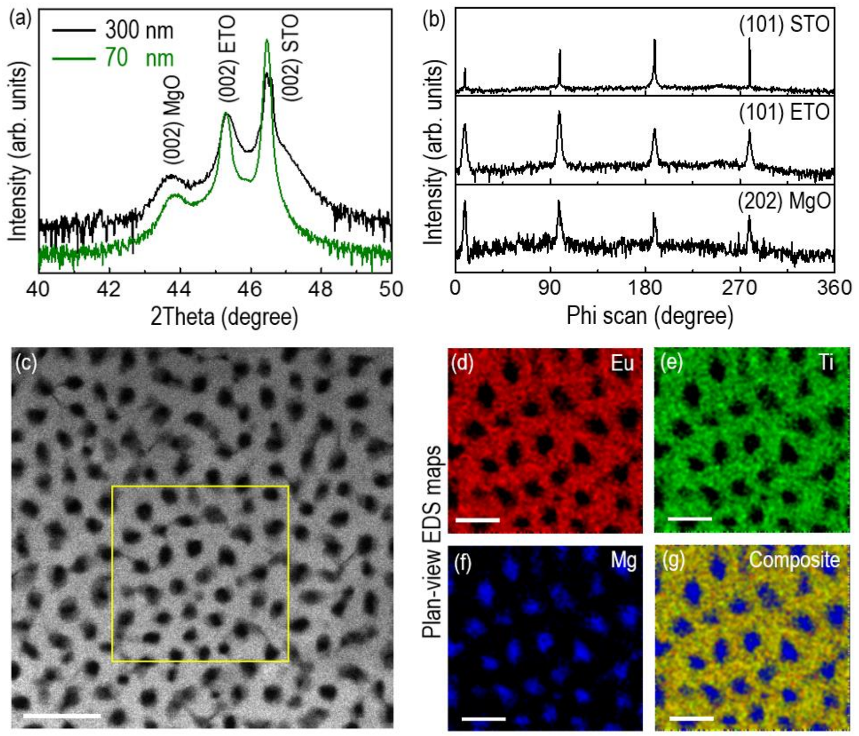

Supplementary Figure 2 Structural analysis by XRD and STEM. (a) XRD θ-2θ detail scans around (002) reflection of ETO:MgO VAN films. (b) Phi scans of (101) STO, (101) ETO, and (202) MgO. (c) HAADF-STEM image of an ETO:MgO VAN film in plan-view orientation, showing MgO nanopillars embedded in an ETO matrix. Scale bar: 20 nm. (d-g) EDS elemental maps of an ETO:MgO VAN film, corresponding to the yellow rectangle region in (c). Scale bar: 10 nm.

Supplementary Table 1

Lattice constants for ETO in bulk, plain ETO film, and ETO in the VAN film

	IP lattice constant (Å)	OOP lattice constant (Å)	Vertical strain	c/a ratio
ETO bulk	3.905	3.905		1
Plain ETO film (300 nm)	3.905	3.905	0	1
ETO in VAN film (70 nm)	3.90±0.01	4.01±0.01	+2.56%	1.03
ETO in VAN film (300 nm)	3.90±0.01	4.01±0.01	+2.56%	1.03

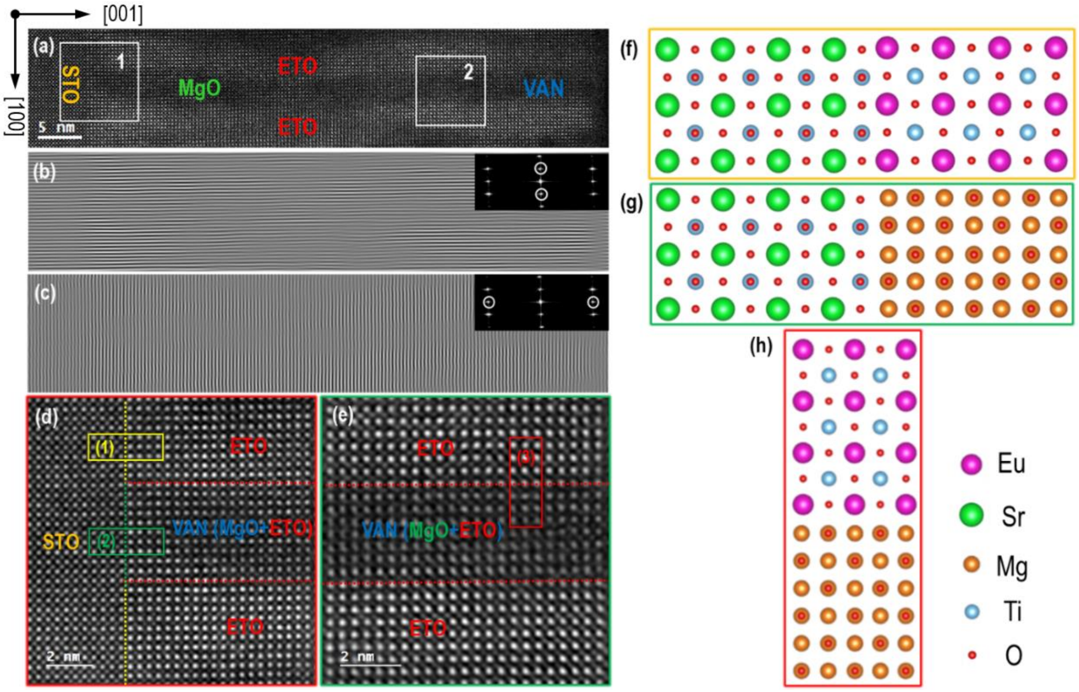

Supplementary Figure 3 STEM investigation of interfacial lattice match. (a) The close-up STEM image of a single MgO nanopillar (dark region) in an ETO:MgO VAN film along the cross-sectional direction. Inverse fast Fourier transform (IFFT) images of (a), showing good lattice match between an ETO:MgO VAN film and a STO substrate both along the IP (b) and OOP (c) directions. (d) and (e) Zoom-in STEM images of regions 1 and 2 highlighted by white boxes in (a), respectively. The lattice matching diagrams for (f) ETO/STO interface, (g) MgO/STO interface, and (h) ETO/MgO interface, corresponding to regions 1 and 2 in (d) and region 3 in (e), respectively. It is worth noting that, because the MgO nanopillars are very narrow with diameters of ca. 4 nm, i.e. smaller than the thickness of the TEM sample (ca. 10 nm), signals from both the ETO and MgO regions were collected in STEM images of VANs along the cross-section direction.

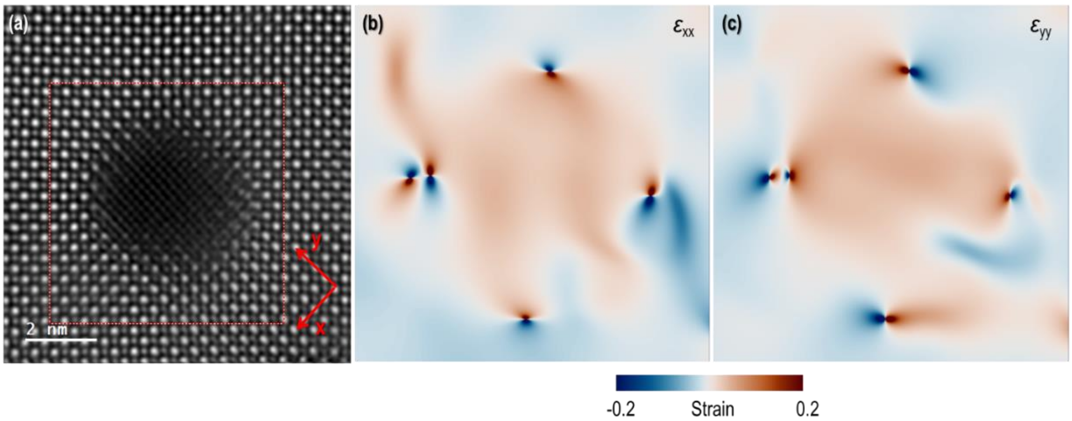

Supplementary Figure 4 Strain analysis by STEM. (a) The close-up STEM image of an individual MgO nanopillar (dark region) along the plan-view direction. (b) and (c) are strain maps ε_{xx} and ε_{yy}. Misfit dislocations are present (pinched regions) which form to relax the misfit strain between the ETO and MgO phases.

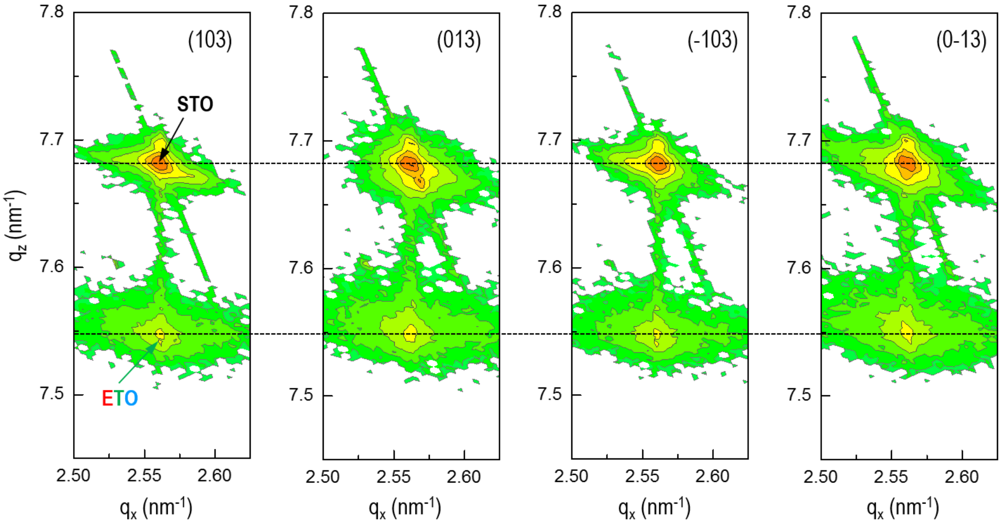

Supplementary Figure 5 Strain analysis by reciprocal space maps. Reciprocal space maps of (103), (013), (10-3), and (0-13) Bragg reflections of STO and ETO for an ETO:MgO VAN film grown on STO substrate.

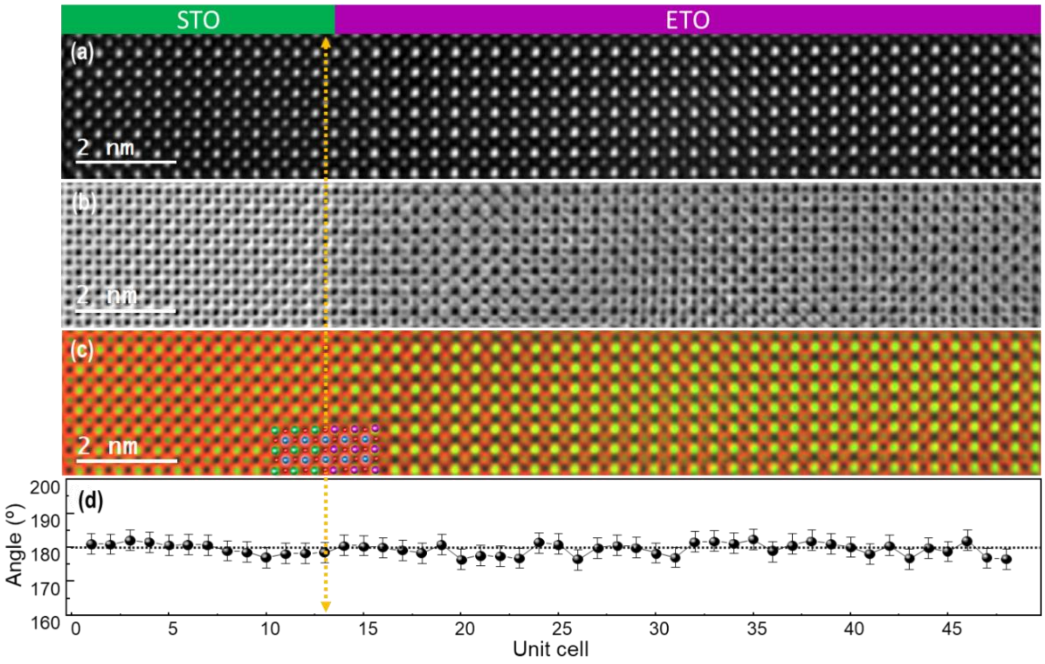

Supplementary Figure 6 STEM investigation of oxygen octahedra. (a) HAADF, (b) ABF and (c) composite image of the ETO phase in the VAN film in cross-sectional orientation. (d) The measured angle between neighboring oxygen octahedra from the STO substrate to the ETO phase.

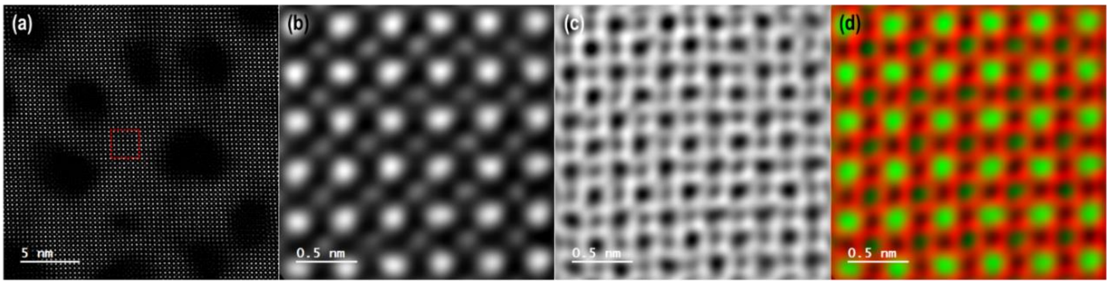

Supplementary Figure 7 STEM investigation of oxygen octahedra. (a) Plan-view HAADF image of an ETO:MgO VAN film. (b) HAADF, (c) ABF and (d) composite images of the ETO phase highlighted by red box in (a). Eu atoms are red and Ti atoms are green.

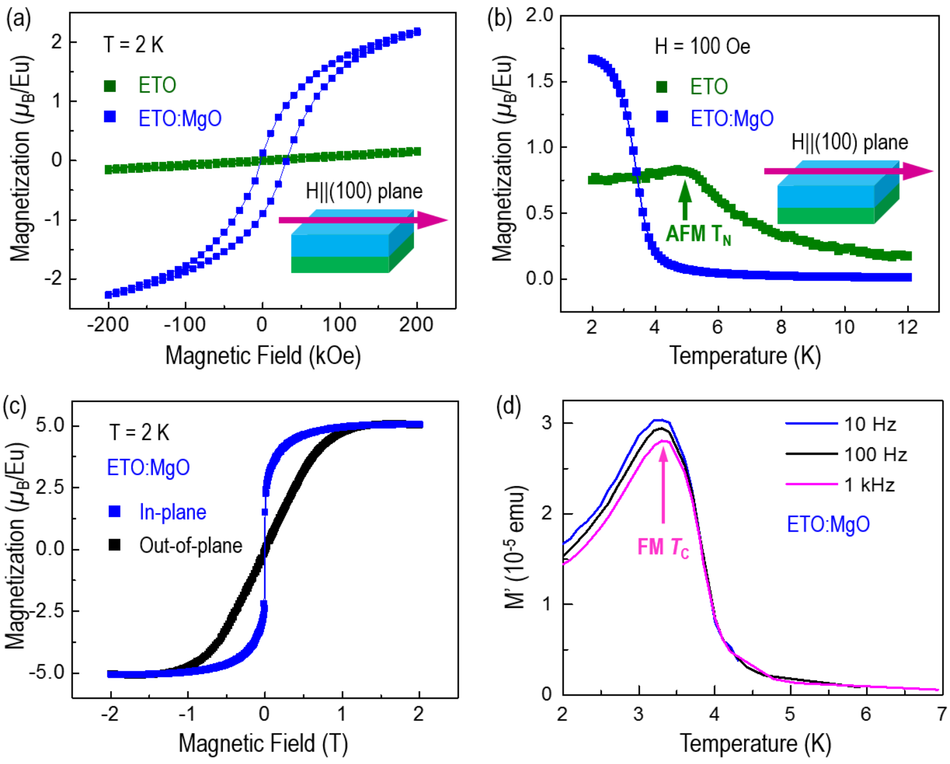

Supplementary Figure 8 Magnetic properties of an ETO:MgO VAN film. (a) Magnetic hysteresis loops of a plain ETO film and an ETO:MgO VAN film measured at 2 K. (b) Temperature-dependent magnetization of a plain ETO film and an ETO:MgO VAN film. (c) Magnetic hysteresis loop of an ETO:MgO VAN film measured by applying a magnetic field along the IP and OOP directions. It can be seen that the magnetic easy axis is along the IP direction. (d) M'-T curves of an ETO:MgO VAN film measured at H_{ac}=5 Oe using the AC frequencies of 10, 100, 1kHz. The arrow indicates the ferromagnetic Curie temperature (T_C) of ~ 3.4 K.

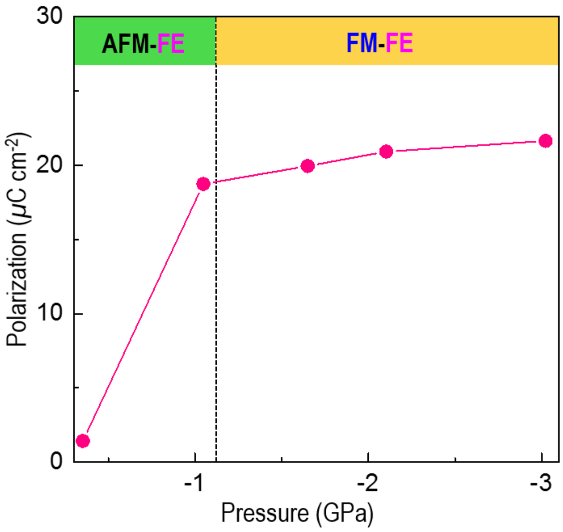

Supplementary Figure 9 Polarization in ETO under negative pressure, from DFT calculations. The calculated ferroelectric polarization of ETO in an ETO:MgO VAN film with the change of the negative pressure.

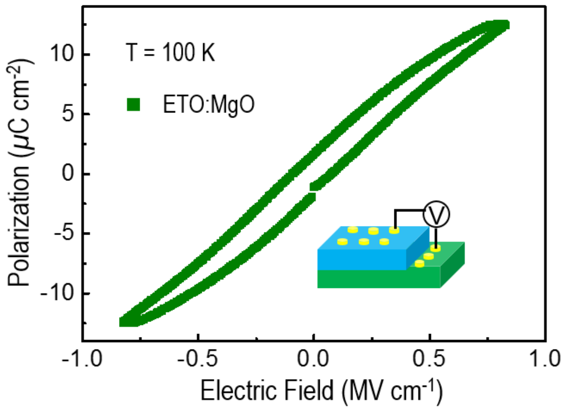

Supplementary Figure 10 Ferroelectric polarization of an ETO:MgO VAN film. Ferroelectric hysteresis loop measured at 1 kHz.

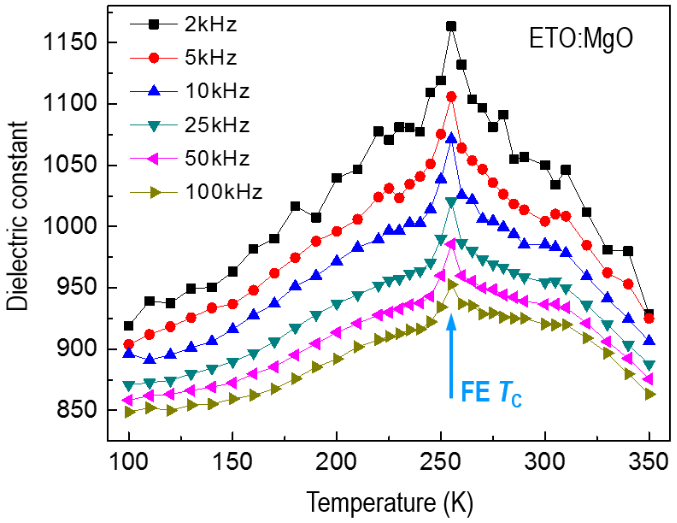

Supplementary Figure 11 Dielectric constant of an ETO:MgO VAN film. Temperature-dependent dielectric constant of an ETO:MgO VAN film measured at different frequencies. The arrow indicates the ferroelectric Curie temperature (T_C) of ~ 255 K.

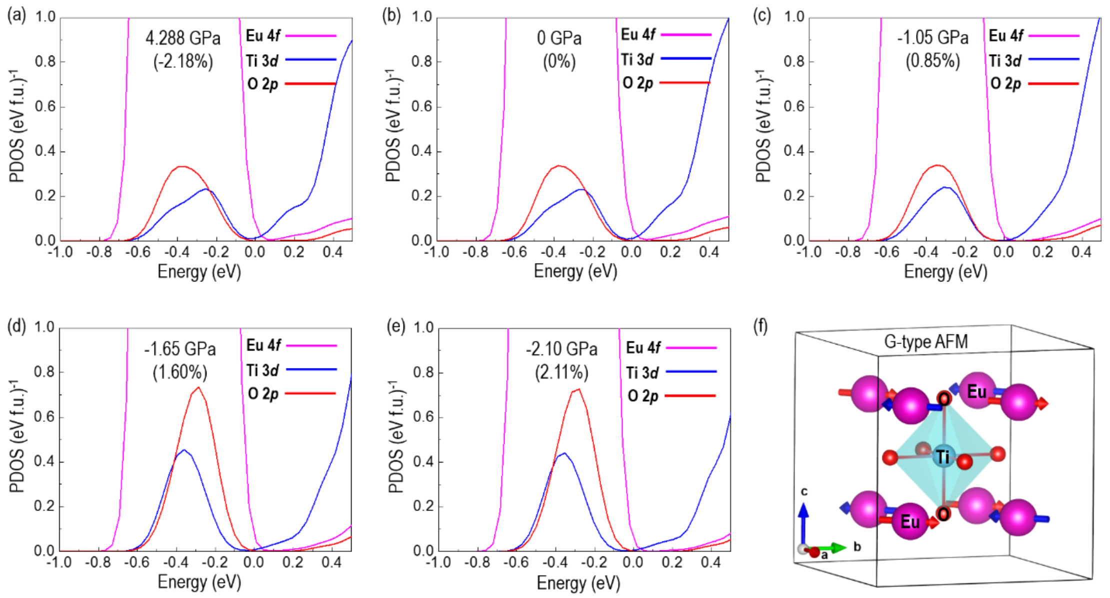

Supplementary Figure 12 Projected density of states in ETO from DFT calculations. (a-e) DFT calculated projected density of states (PDOS) of ETO under different negative pressures (vertical strain). (f) Schematic diagram of ETO with G-type AFM magnetic configuration.

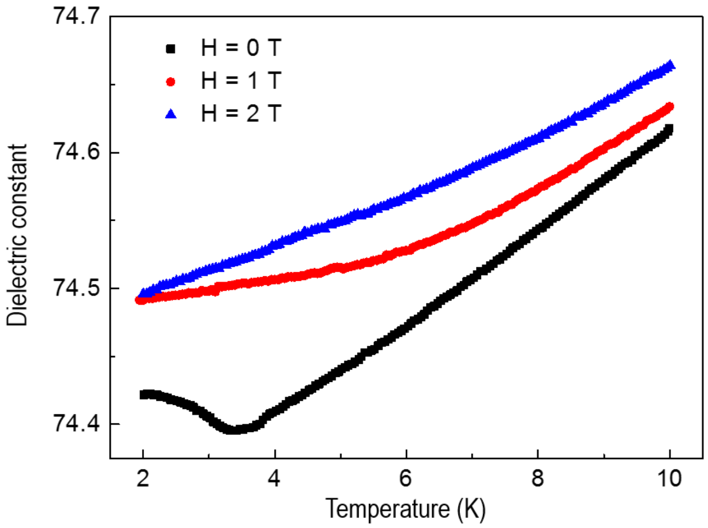

Supplementary Figure 13 Magnetodielectric coupling of an ETO:MgO VAN film. Temperature dependence of dielectric constant measured at different magnetic fields and 100 kHz.

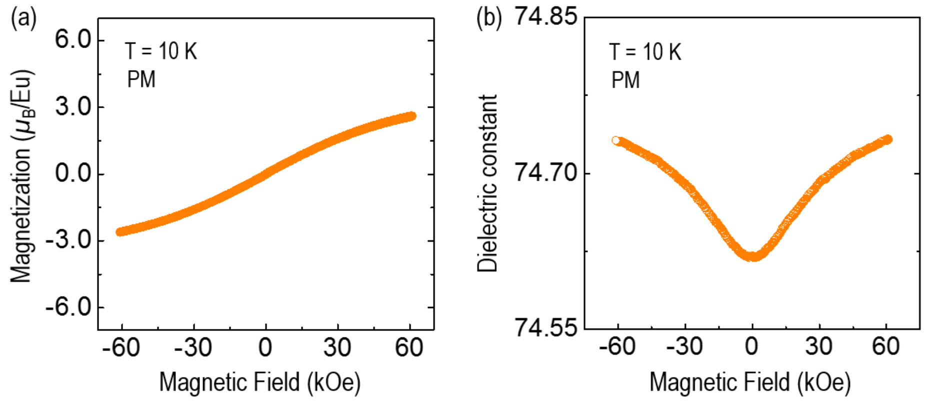

Supplementary Figure 14 Magnetodielectric coupling of an ETO:MgO VAN film. Magnetic hysteresis loop (a) and magnetic field-dependent dielectric constant (b) of an ETO:MgO VAN film measured at 10 K.

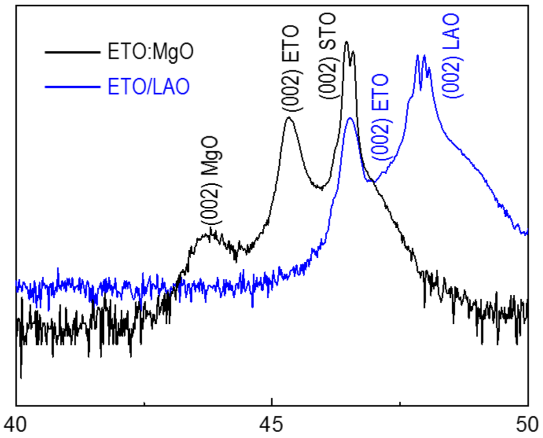

Supplementary Figure 15 Structural analysis by XRD. Comparison of XRD θ-2θ detail scans around (002) reflection of an ETO:MgO VAN film grown on a STO substrate and a plain ETO film grown on a LAO substrate.

Supplementary Discussion 1

Inspired by the fact that the external magnetic field may have substantial effect on the lattice parameter of the ETO[1], which leads to a magnetostriction, we proposed an energy term related to the magnetic field induced strain in addition to the biquadratic coupling term of the spins and phonon (lattice). For simplicity, we only consider the linear coupling between the strain and the magnetic field through the real behavior of strain under magnetic field is more complicated, as was reported in literature[2].

The free energy of the ETO can be expressed as:

$$F(M,P)=F_0+\frac{1}{2}A_{p0}(T-T_{CP})P^2+\frac{1}{4}B_pP^4+\frac{1}{2}A_MM^2+\frac{1}{4}B_MM^4-\frac{1}{2}\lambda M^2P^2-\frac{1}{2}\alpha\sigma HP^2 \qquad (1)$$

Here, F_0 is the free energy of the PM-PE state, T is the temperature, and T_{CP} is the FE transition temperature in the absence of the external magnetic field. The polarization P and magnetization M are the ferroelectric and ferromagnetic order parameters, respectively. A_{p0}, B_p, A_M and B_M are the expansion coefficients. It should be noted that λ is the strength of the biquadratic spin-lattice coupling coefficient and is negative for ferromagnetic order[3]. The term of $-\frac{1}{2}\alpha\sigma HP^2$ is introduced to describe the influence from the magnetic field induced strain, where H is the external magnetic field, σ is the effective strain imposed on the ETO, and α is the corresponding coefficient to scale the strength of the tuning effect of the magnetic field on the lattice parameters.

By dealing with the free energy according to the thermodynamic analysis, we can obtain the reciprocal of dielectric susceptibility as following:

$$\chi^{-1}=A_{p0}(T-T'_{CP})+3B_pP^2 \qquad (2)$$

where $T'_{CP}=T_{CP}+\frac{\lambda M^2}{A_{p0}}+\frac{\alpha\sigma H}{A_{p0}}$. Since the T_{CP} is much higher than the T_{CM}, both the spontaneous polarization P_s and spontaneous magnetization M_s are zero in the absence of the magnetic field for $T>T_{CP}$. And consequently, χ, according to Eq. (2),

follows the Curie-Weiss Law, i.e. $\chi = \frac{C}{T - T_{CP}}$, with the Curie-Weiss constant $C = \frac{1}{A_{p0}}$. For $T_{CM} < T < T_{CP}$ and $H = 0$, the spontaneous magnetization M_S is zero, while the spontaneous polarization P_S is nonzero. The ETO system is in its PM-FE state and the dielectric constant, according to Eq. (2), can be expressed as $\chi_{(\lambda=0)} = \frac{1}{2(T_{CP} - T)}$, where $\chi_{(\lambda=0)}$ is the dielectric susceptibility in the ferroelectric phase without the coupling between the ferroelectric and magnetic orderings.

At temperatures lower than T_{CM}, the ETO system is in its FM-FE state, i.e. both M_S and P_S are nonzero and these two order parameters are coupled to each other. As a result, the dielectric susceptibility in the absence of the external magnetic field (H =0) can be written as below, where $\chi_{(\lambda=0)}$ means the dielectric susceptibility in the ferroelectric phase without the coupling between the ferroelectric and magnetic orderings.

$$\chi = \frac{\chi_{(\lambda=0)}}{1 + 2\lambda \chi_{(\lambda=0)} M^2} \approx \chi_{(\lambda=0)}(1 - 2\lambda \chi_{(\lambda=0)} M^2) \qquad (3)$$

As far as the magnetostriction is concerned, the dielectric susceptibility for the FM-FE state in ETO can be obtained as:

$$\chi = \frac{\chi_{(\lambda=0)}}{1 + 2\lambda \chi_{(\lambda=0)} M^2 + 2\alpha \chi_{(\lambda=0)} H\sigma} \approx \chi_{(\lambda=0)}(1 - 2\lambda \chi_{(\lambda=0)} M^2 - 2\alpha \chi_{(\lambda=0)} H\sigma) \qquad (4)$$

where λ is negative and σ is positive.

Supplementary Discussion 2

According to the previous investigation on the spin-phonon coupling of bulk ETO, a strong coupling between the Eu spins and dielectric properties was realized through a modified T_{1u} phonon mode, confirmed by an abrupt changed dielectric constant under different magnetic fields. Meanwhile, the magnitude of the phonon frequency (ω_0) change with an applied magnetic field can be estimated from that of the dielectric-constant change[4]. In our results, we presented the dielectric constant under the different magnetic fields as shown in Fig. 6(a). The dielectric constant shows a monotonic increasing trend and saturate as the magnetic field increases at the lower range. Hence, we can assume that the increase of ε by 0.09% under a magnetic field means a decrease of ω_0 by 0.0045%, calculated by the formula as below:

$$\varepsilon(\omega) = \varepsilon_1(\omega) - \mathrm{i}\varepsilon_2(\omega) = \varepsilon_\infty + \frac{4\pi N \mathrm{e}^{*2}/\mu}{\left(\omega_0^2 - \omega^2\right) + \mathrm{i}\Gamma\omega} \tag{5}$$

where N is the number of unit cells per volume, e^* is the effective charge of ions, μ is the effective mass of ions, ω_0 is the phonon frequency, and Γ is the scattering rate of the phonon.

Focused on the ETO phase, the spin-phonon coupling can be simply described as the configuration of Eu spins under lower magnetic field varies the frequency of a phonon mode, *via* the changed hybridization between the Eu 4*f*, O 2*p* and Ti 3*d* orbitals[4]. For the magnetostriction, the previous theory confirmed that a tiny striction stemming from the rearrangement of Eu spins under a higher magnetic field also influence the frequency of a phonon mode[1].

Based on the relative length change in the ETO phase under various magnetic field[5], we systematically investigated the competition between the two mechanisms, i.e. spin-phonon coupling and magnetostriction. Firstly, a typical spin-phonon coupling effect dominates at a lower temperature, monotonically increasing dielectric constant. Then, considering the magnetostriction effect, a reversed trend in the dielectric constant is dependent on the increased length. Meanwhile, with the decreased temperature, the

strengthened ability of magnetic induced strain caused to an enhanced magnetostriction effect. Lastly, an abnormal magnetostriction of the crossover length leads to the decreased in the coefficient α under a higher magnetic field, showing a weakened magnetostriction effect.

Supplementary Discussion 3

The magnetic exchange constants were calculated by mapping the total energy difference between different magnetic configurations onto the Heisenberg Hamiltonian $H = -2\sum_{ij} J_{ij} S_i S_j$, where J_{ij} is the exchange constant of magnetic interactions between the Eu 4*f* spins at the *i* and *j* sites and S_i is the spin vector at the *i* site. The total energies for different magnetic configurations can be described with Heisenberg Hamiltonians below.

$H_{A\text{-}AFM}=J_0+4|S|^2(-8J_{11}+4J_{12}-8J_{21}+16J_{22}+16J_3)$

$H_{FM}=J_0+4|S|^2(-8J_{11}-4J_{12}-8J_{21}-16J_{22}-16J_3)$

$H_{G\text{-}AFM}=J_0+4|S|^2(8J_{11}+4J_{12}-8J_{21}-16J_{22}+16J_3)$

$H_{C\text{-}AFM}=J_0+4|S|^2(8J_{11}-4J_{12}-8J_{21}+16J_{22}-16J_3)$

$H_{D\text{-}AFM}=J_0+2|S|^2(0J_{11}-0J_{12}+16J_{21}+0J_{22}+0J_3)$

$H_{E\text{-}AFM}=J_0+2|S|^2(0J_{11}+8J_{12}+0J_{21}+0J_{22}-0J_3)$

$H_{F\text{-}FiM}=J_0+2|S|^2(0J_{11}-0J_{12}+0J_{21}-0J_{22}+32J_3)$

$H_{H\text{-}FiM}=J_0+2|S|^2(0J_{11}-8J_{12}+0J_{21}-0J_{22}+0J_3)$

References

1. Reuvekamp P, *et al.* Tiny cause with huge impact: polar instability through strong magneto-electric-elastic coupling in bulk $EuTiO_3$. *Journal of Physics: Condensed Matter* **27**, 262201 (2015).
2. Kamba S, *et al.* Magnetodielectric effect and phonon properties of compressively strained $EuTiO_3$ thin films deposited on (001)$(LaAlO_3)_{0.29}(SrAl_{1/2}Ta_{1/2}O_3)_{0.71}$. *Physical Review B* **85**, 094435 (2012).
3. Lee JH, *et al.* A strong ferroelectric ferromagnet created by means of spin–lattice coupling. *Nature* **466**, 954-958 (2010).
4. Katsufuji T, Takagi H. Coupling between magnetism and dielectric properties in quantum paraelectric $EuTiO_3$. *Physical Review B* **64**, 054415 (2001).
5. Reuvekamp P, *et al.* Spin-lattice coupling induced crossover from negative to positive magnetostriction in $EuTiO_3$. *Physical Review B* **90**, 094420 (2014).
